# Genome-Wide Identification and Heat Stress-Induced Expression Profiling of the *Hsp70* Gene Family in *Phoebe bournei*

**DOI:** 10.3390/biology14060602

**Published:** 2025-05-25

**Authors:** Yiming Lin, Yan Jiang, Zhuoqun Li, Yuewang Niu, Chenyu Gong, Xin He, Shipin Chen, Shijiang Cao

**Affiliations:** College of Forestry, Fujian Agriculture and Forestry University, Fuzhou 350002, China; 13606999239@163.com (Y.L.); jyan2023@126.com (Y.J.); 13285946066@163.com (Z.L.); 13463995879@163.com (Y.N.); 18759319971@163.com (C.G.); 5220422102@fafu.edu.cn (X.H.);

**Keywords:** *Hsp70* gene family, *Phoebe bournei*, genome-wide identification, heat stress

## Abstract

*Phoebe bournei*, a rare tree species native to China, holds considerable economic importance. The heat shock protein 70 (Hsp70) family is a group of molecular chaperones that is broadly distributed across living organisms and play a critical role in processes like growth, development, and stress response. While *Hsp70* genes have been identified and studied in various plant species, their specific functions in the growth and development of *P. bournei* remain unexplored. We performed a comprehensive analysis of the *Hsp70* gene family in *P. bournei*, identifying a total of 45 *Hsp70* genes, which were classified into four groups (I–IV) through phylogenetic analysis. All Hsp70 proteins possessed conserved structural domains, including motif 7, and introns were present in 77.8% of the genes. Chromosomal localization and collinearity analyses of the *Hsp70* genes revealed their evolutionary relationships and potential gene duplication events. Examination of the cis-acting elements within the Hsp70 promoter regions revealed that the predominant elements were associated with growth and development, followed by those responsive to hormones, and then elements linked to abiotic stress. Nine genes with high expression were selected for RT-qPCR analysis. Under high-temperature stress, all nine genes were differentially upregulated, and most of these genes belonged to subfamilies II and III, indicating that these two subfamilies have strong potential for heat resistance. In this study, we have elucidated the molecular characteristics and heat response properties of the *Hsp70* gene family in *P. bournei*, revealing the mechanisms behind its heat stress response. Our work provides a reference for stress breeding in *P. bournei* and a theoretical basis for the exploration of heat tolerance in woody plants.

## Simple Summary

Heat stress is one of the important factors affecting the distribution of *Phoebe bournei*, we analyzed *Hsp70* in *P. bournei*, encompassing physicochemical properties, gene structure, phylogenetic tree construction, collinearity analysis, and cis-elements. Forty-five *PbHsp70s* were identified and classified into four groups (I–IV). Specifically, this study systematically demonstrates the molecular characteristics of *PbHsp70* genes in *P. bournei*, which contributes to broadening our understanding of the evolutionary relationships. Moreover, the response of *PbHsp70* genes to heat stress was studied, and a set of candidate genes for heat stress resistance was provided, which provided new insights for further investigating the role of Hsp70 proteins and for stress breeding.

## 1. Introduction

The heat shock transcription factor (HSF) family responds to biotic and abiotic stresses, enhancing plants resistance and supporting normal growth and development [1]. Temperature is a fundamental environmental factor influencing plant growth and development. It strongly affects physiological and metabolic processes in plants; therefore, extensive research efforts have focused on elucidating the adaptive mechanisms of plants under thermal stress [2,3]. Thermotropic stress induced through heat shock treatment activates plants’ endogenous proteinaceous defense networks, significantly upregulating the expression of heat shock proteins (Hsps), which are key components of the cellular stress response mechanism [4]. Based on the protein size (60, 70, 90, and 100 kDa), Hsps are categorized into subgroups, namely the *Hsp60*, *Hsp70*, *Hsp90,* and *Hsp100* gene families [5]. Among these heat shock protein families, Hsp70 is known as the major heat shock protein and consists of two functional domains, the nucleotide-binding domain (NBD) and the substrate-binding domain (SBD) [6]. Hsp70 is highly conserved and plays a crucial role in sustaining plant growth homeostasis under heat stress at sublethal temperatures [7,8,9]. Hsp70 acts as a molecular chaperone in the presence of temperature stress by assisting in restoring proper protein folding and repairing denatured proteins to achieve plant protection [10]. The Hsp70 molecular chaperone system has shown significant gene expansion during plants’ evolution [11]. Genome-wide analyses have revealed substantial interspecies variation in the number of family members; in particular, *Arabidopsis thaliana* (17 genes), *Oryza sativa* (26 genes), *Capsicum annuum* L. (21 genes), and *Glycine max* (L.) Merr. (61 genes) have been systematically characterized [9,12]. In *A. thaliana*, Hsp70 loss-of-function mutant plants exhibited stunted growth and an abnormal leaf morphology [13], and double-allele knockout experiments have confirmed the critical regulatory role of Hsp70 in gametophyte development [14]. In addition, Hsp70 proteins located in the cytoplasm and nucleus play key regulatory roles in *A. thaliana* plant development under abiotic stress, such as salt damage and drought [15,16].

*Phoebe bournei* is a rare evergreen tree species that is endemic to China; it is classified as a key protected wild plant species due to the high economic and cultural value of its timber. However, with global warming, high-temperature stress has become a key environmental issue threatening the survival of *P. bournei* populations [17,18]. Studies have shown that *P. bournei*, which is naturally distributed in subtropical humid climate zones, is unable to adapt to extremely high temperatures. High-temperature stress inhibits photosynthesis, destroys the cell membrane system, and accelerates the accumulation of reactive oxygen species, leading to growth inhibition and even seeding mortality [19,20]. In this context, it is crucial to analyze the regulation mechanism of the *Hsp70* gene family expression in *P. bournei* under heat stress [21,22]. However, to date, a systematic genome-wide survey and comprehensive functional analysis of this gene family in *P. bournei* remain unexplored.

In this study, we present the first genome-wide investigation of the *Hsp70* gene family in *P. bournei*. Through comprehensive bioinformatics analyses, we identified gene family members, reconstructed their phylogenetic relationships and collinearity patterns, and predicted their protein structures using established computational tools [23]. In addition, the expression of the proteins was analyzed via real-time fluorescence quantitative PCR (RT-qPCR) to ascertain their reactions to heat shock stress in *P. bournei* [24]. This study aims to provide important genetic information for the *Hsp70* gene family of *P. bournei* and offer high-quality reference genes for the genetic engineering and breeding of *P. bournei.* This, in turn, will provide important support for improvements in the stress tolerance of rare tree species.

## 2. Materials and Methods

### 2.1. Data Acquisition and Sample Collection

The whole-genome data of *P. bournei* (CNSA: CNP0002030) were obtained from the China National GeneBank Database (CNSA, accessed on 17 January2025) [25]. The genome sequence files of *A. thaliana* and *P. trichocarpa* were acquired from the Arabidopsis Information Resource (TAIR, https://www.arabidopsis.org/, accessed on 17 January 2025) and Phytozome v13 (https://phytozome-next.jgi.doe.gov/, accessed on 17 January 2025) [26,27]. After plant material preparation and sampling, 1-year-old tissue-cultured *P. bournei* seedlings cultivated in artificial climate chambers were selected as the experimental materials. Seedlings that exhibited uniform growth were divided into experimental groups and control groups and subjected to stress treatments. Each treatment consists of three biological replicates, following standardized protocols for plant material collection. We ensured that all plants were in the same growth state and under the same ecological conditions. The instruments were sterilized and disinfected. Three to five healthy leaf slices were randomly selected from each *P. bournei* plant. The samples were rinsed three times with pure water, and the surface water was blotted with sterile filter paper. Following treatment, leaf samples were cryo-preserved through rapid immersion in liquid nitrogen and subsequently stored at −80 °C to preserve RNA integrity prior to extraction.

### 2.2. Characterization and Functional Analysis of Hsp70 Gene Family

The amino acid sequence of the Hsp70 protein from *A. thaliana* was used as a probe to perform a local BLAST search, followed by the reverse BLAST comparison of the obtained sequences using NCBI (https://www.ncbi.nlm.nih.gov/, accessed on 20 January 2025) [28]. To further identify Hsp70 in *P. bournei*, we used a Hidden Markov Model (HMM) profile of Hsp70 (PF00012) from the Pfam 37.2 database (accessed on 20 January 2025) for analysis, using HMMER-3.2.1 (http://hmmer.org/download.html, accessed on 20 January 2025) with an e-value < 10^−5^ and other parameters set to default values [29]. We then utilized ExPASy 3.0 (https://www.expasy.org/, accessed on 21 January 2025) for the evaluation of the protein attributes [30]. A protein is classified as basic when its isoelectric point (pI) exceeds 7, whereas acidic characterization occurs when the pI falls below this threshold [31]. The instability index less than 40 indicates that the protein is stable [32]. Moreover, a comprehensive analysis of the potential subcellular localization of the Hsp70 proteins in *P. bournei* through WoLF PSORT (https://wolfpsort.hgc.jp/, accessed on 22 January 2025) facilitates better exploration of the functions of these proteins [33].

### 2.3. Phylogenetic Analysis of Hsp70

The Hsp70 protein sequences from *A. thaliana*, *P. bournei,* and *Populus trichocarpa* were aligned using the Muscle program. Subsequently, a phylogenetic tree was constructed using the IQ-TREE software (https://iqtree.github.io/, accessed on 23 January 2025) to elucidate their evolutionary relationships [34,35]. We constructed phylogenetic trees using the maximum likelihood (ML) method through the Muscle program, with the default settings [36]. The bootstrap method was performed with 1000 replicates. The iTOL online platform (https://itol.embl.de/, accessed on 23 January 2025) was used to visualize and annotate the phylogenetic trees to enhance clarity and interpretability [37].

### 2.4. Motifs and Gene Structures Analysis

The NCBI’s CDD tool was used to predict the inherent conserved structural domains of the *Hsp70* family in *P. bournei* [38]. In addition, the conserved motifs of the *Hsp70* family in *P. bournei*, *A. thaliana,* and *P. trichocarpa* were analyzed using the MEME 5.5.7 (https://meme-suite.org/meme/tools/meme, accessed on 23 January 2025) [39]. The MEME analysis was performed using the default settings, adjusting the maximum number of motifs to 10. Sequentially, the generated output was used to construct an overall comparative map including the ML phylogenetic tree. The phylogenetic trees, motif conservation profiles, and gene structural annotations were computationally integrated and visually rendered.

### 2.5. Chromosomes Localization and Collinearity Analysis of Hsp70s

The gene sequence and annotation data of *P. bournei* were used to visualize the *Hsp70* family’s chromosome positions using the TBtools v2.210 software [40]. The genomic information of *P. bournei* was obtained from the NCBI database, and its *Hsp70* gene family was analyzed for collinearity. For the collinearity analysis, we utilized the MCScanX from TBtools software [41].

### 2.6. Cis-Acting Elements Analysis of Hsp70

To investigate the transcriptional regulation of the *Hsp70* family in *P. bournei*, we extracted the 2000 bp upstream sequence of the Hsp70 promoter and subsequently analyzed this region using PlantCARE (https://planttfdb.gao-lab.org/index.php?sp=Ath, accessed on 25 January 2025) to predict the occurrence of *cis*-elements [42]. After screening and classification, Cytoscape 3.10.3 was applied to visualize the data [43].

### 2.7. Analysis of Expression, RNA Extraction and RT-qPCR

We used the FastPure Plant Total RNA Isolation Kit (for polysaccharide- and polyphenol-rich tissue) (Vazyme Biotech Co., Ltd., Nanjing, China) to extract RNA from differently treated *P. bournei* leaves. Using the RSEM v1.2.8 tool, the gene expression levels of individual samples were quantified with high precision through computational analysis. From this, we obtained the fragments per kilobase of transcripts per million fragments (FPKM) values [44]. Based on the calculated FPKM values, detailed heatmaps were generated using TBtools software to visualize the gene expression levels and make the results clearer and more concise [40].

Using the First-Strand cDNA Synthesis Mix#F022 (Beijing Lablead, Beijing, China), reverse transcription was carried out using a method designed to prevent potential genomic DNA contamination and improve cDNA synthesis efficiency. The 2x Realab Green PCR Fast Mixture R0202 was used for the RT-qPCR analysis. Each sample was analyzed with three replicates. The experimental setup consisted of a 96-well plate with 20 µL of the reaction system per well, consisting of 10 µL Taq SYBR^®^ Green qPCR Premix, Beijing, China, 0.4 µL each of the positive and negative primers, 2 µL cDNA template, and 7.2 µL nuclease-free water. The reference gene primers were designed based on previously published studies [45]. PbEF1α was selected as the reference gene. The primer design and the synthesis of the target gene were completed by Sangon Biotech (Shanghai) Co., Ltd., Shanghai, China. The PCR amplification was executed using a four-step thermal cycling protocol. Forty reaction cycles were performed. The relative expression levels of the target genes were evaluated through the implementation of the 2^−ΔΔCT^ method and the GraphPad Prism 7.0 software. Table S1 shows the qRT-PCR primers.

## 3. Results

### 3.1. Identification and Analysis of Hsp70 Proteins

The Hsp70 proteins of *P. bournei* were identified using BLAST and HMMER-3.2.1 (https://blast.ncbi.nlm.nih.gov/Blast.cgi; http://hmmer.org/download.html, accessed on 20 January 2025). In total, 45 PbHsp70 proteins were identified sequentially from top to bottom in *P. bournei* using the chromosomal positions. The amino acid (AA) count varied from 114 aa (*PbHsp70-24*) to 1358 aa (*PbHsp70-42*), with an average length of 457 aa. The grand average of hydropathy (GRAVY) of the *Hsp70* family ranged from −1.119 (*PbHsp70-10*) to −0.306 (*PbHsp70-21*), indicating that the GRAVY scores for the entire family were below 0, suggesting that all Hsp70 proteins were hydrophilic. The theoretical isoelectric point (pI) ranged from 4.65 (*PbHsp70-43*) to 9.75 (*PbHsp70-37*). The isoelectric point (pI) values of 28 Hsp70 proteins were less than 7.0, indicating acidity, while the remaining 17 had pl values above 7.0, indicating basic isoelectric points. The molecular weights (MWs) of the 45 Hsp70 proteins ranged from 12.93 kDa (*PbHsp70-21*) to 150.81 kDa (*PbHsp70-42*), with an average MW of 50.92 kDa. The aliphatic index (AI) ranged from 67.72 (*PbHsp70-24*) to 103.21 (*PbHsp70-19*). The instability index (II) ranged from 23.18 (*PbHsp70-35*) to 56.09 (*PbHsp70-11*), indicating that most Hsp70 proteins were unstable. Moreover, subcellular localization predictions showed that the Hsp70 proteins in *P. bournei* were mainly localized to the cytoplasm (Table 1).

### 3.2. Phylogeny and Classification of Hsp70s

**Figure 1:**
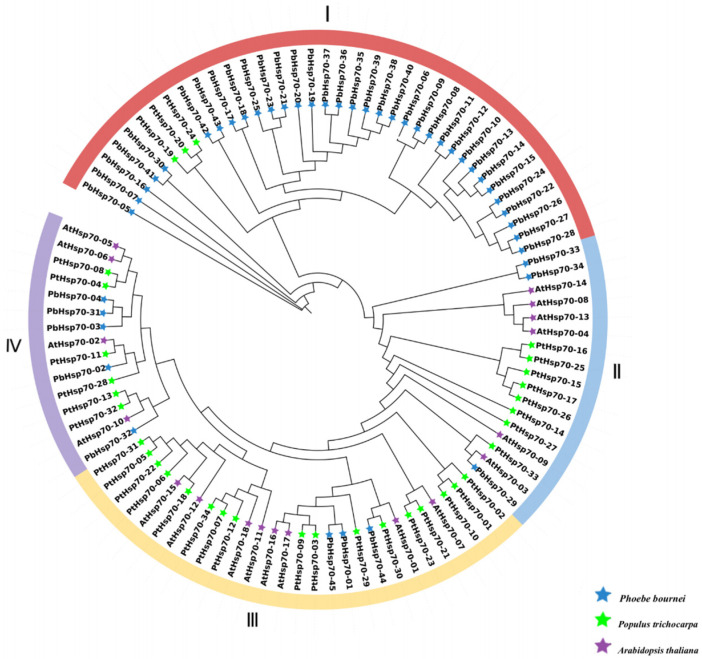
Phylogenetic tree generated for 97 Hsp70 proteins retrieved from *P. bournei*, *A. thaliana,* and *P. trichocarpa*. The phylogeny test was performed using the bootstrap method with 1000 replications.

Based on the ML method, the phylogenetic relationship was constructed based on a total of 97 Hsp70 protein sequences from *P. bournei*, *P. trichocarpa,* and *A. thaliana* (Figure 1). Compared to the *AtHsp70* family in *A. thaliana*, based on the overall morphology of the evolutionary tree, the *Hsp70* gene family in *P. bournei* showed a more distinct branching structure, which was roughly divided into four major regions (I–IV), with subfamily I having the largest number of members (34 species) and family IV having the smallest (15 species). This may reflect the different evolutionary branches or the functional differentiation of gene families during the evolutionary process. Gene members within each region are closely related, while genes between different regions are more divergent. The distribution of the *Hsp70* genes in all regions of *P. bournei* indicates that the *Hsp70* family in this species is highly diverse and may have undergone gene exchange with the *Hsp70* genes of other species, or they may have had shared ancestors during the evolutionary process. Compared with other regions, subfamily I is relatively independent in its gene composition and may represent a relatively unique evolutionary branch of the *Hsp70* gene family in *P. bournei*. The *Hsp70* genes of *A. thaliana* and *P. trichocarpa* were clustered with some genes of *P. bournei*, indicating that these plants are evolutionarily related, and the *Hsp70* gene family may have conserved certain structural and functional elements among different plant species in response to similar biological processes, such as cellular stress responses. From the branching of the evolutionary tree, it was observed that some genes appeared in clusters, which may indicate gene duplication events. After gene duplication, the newly generated copies may experience different evolutionary pressures, leading to functional divergence. In some branches, the genes may gradually adapt to different environmental conditions or cellular physiological demands, leading to the evolution of Hsp70 proteins with different properties, being able to engage in biological processes including critical processes like protein biosynthesis and quality control mechanisms that maintain cellular homeostasis.

### 3.3. Hsp70 Structure and Motif Analysis

**Figure 2:**
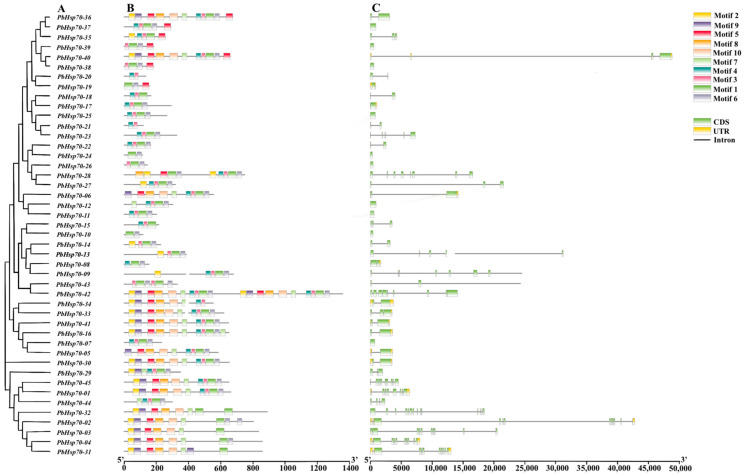
*P. bournei Hsp70* gene family motif and gene structure analysis. (**A**) Phylogenetic relationship of 45 *Hsp70* genes; (**B**) conserved motifs of Hsp70 proteins; (**C**) structure of Hsp70 proteins. Comparative mapping of phylogenetic trees and conserved protein motifs.

**Figure 3:**
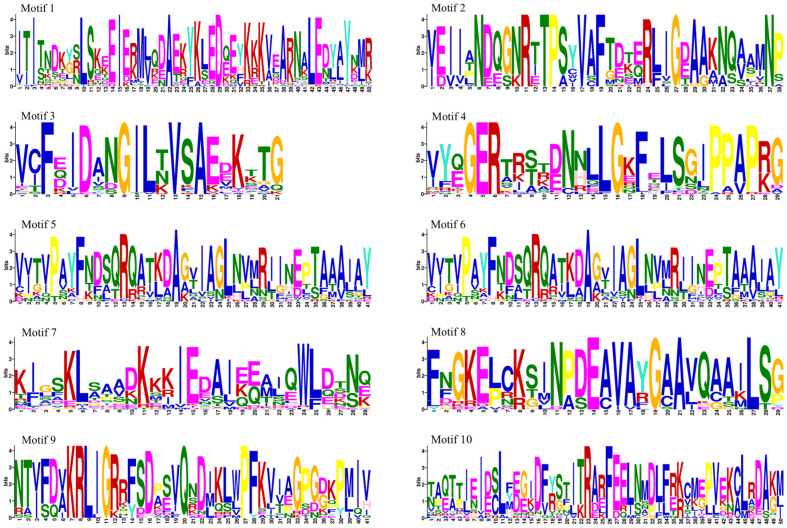
Sequence information for base sequences 1 to 10. The conserved sequence patterns of the *Hsp70* gene were analyzed using the MEME 5.5.7 Suite online software.

The results indicated that most of the conserved motifs were found in the C-terminal structural domain, which participates in protein biosynthesis and functional regulation, and the sequence of the designated motifs started from 2, followed by 9, 5, 8, 10, 7, 4, 3, 1 and, finally, 6 (Figure 2B). Motif 7 was present in all Hsp70 proteins within the structural domain; thus, it may be the active region responsible for the gene’s function (Figure 2C). The conserved motifs within the same subfamily were similar, and motifs 3 and 4 were not present in subfamily IV. In addition, a complex analysis of the intron–exon structure of the *Hsp70* genes was performed to aid in their characterization. Among the 45 *Hsp70* genes, most of them contained introns (77.8%), with some having between one and five (Figure 2C). A total of 13.3% of *Hsp70* genes had two exons and one intron. A survey of the 45 Hsp70 proteins revealed that the number of introns varied between 0 and 13, while the number of exons ranged from 1 to 14. Figure 3 shows the details of sequences 1–10, respectively.

### 3.4. Chromosomal Localization, Collinearity Analysis, and Promoter Analysis of Hsp70s

**Figure 4:**
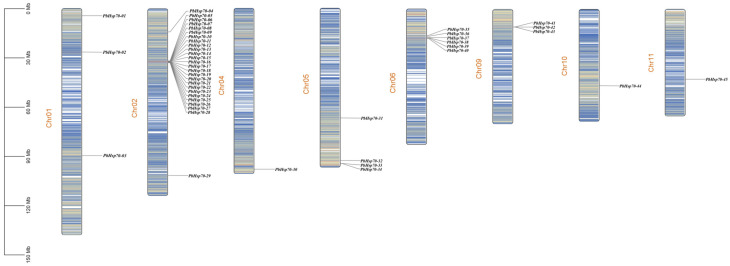
Distribution of *Hsp70* on the chromosomes of *P. bournei.* Chromosome names are labeled in orange, and black indicates allelic locus names.

The chromosomal localization analysis found that the 45 *Hsp70* genes were located on eight chromosomes, and the largest number of *Hsp70* genes was distributed on chromosome 2, with a total of 25 genes, followed by a total of six in Chr06 (Figure 4). Moreover, 12 pairs of tandem duplicated genes were identified in the chromosomes. These were close to each other in the chromosomes and formed clusters on the phylogenetic tree, suggesting that they share similar functions.

**Figure 5:**
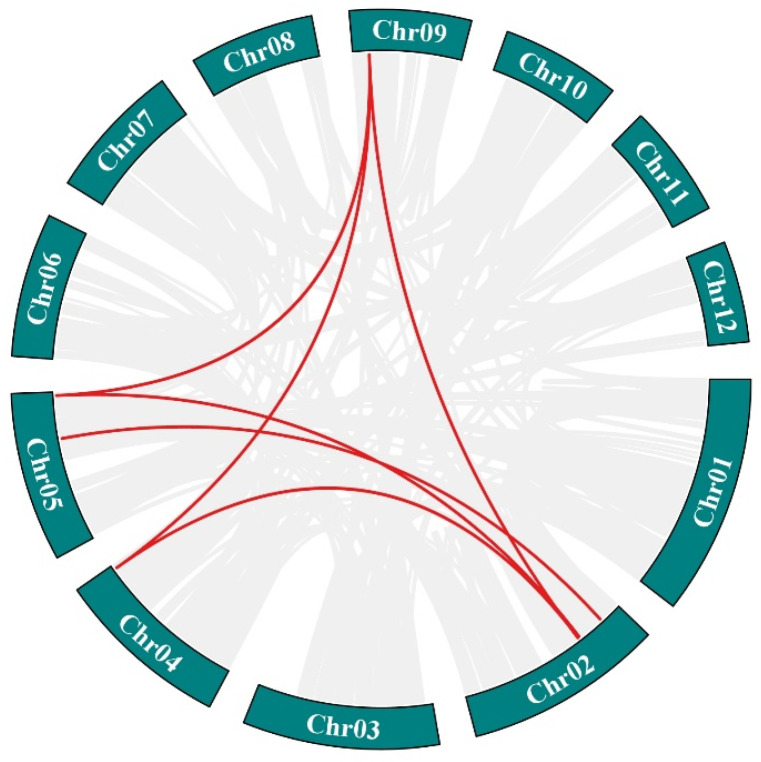
Interspecific covariance in *P. bournei*. Red lines represent segmental duplicate gene pairs.

**Figure 6:**
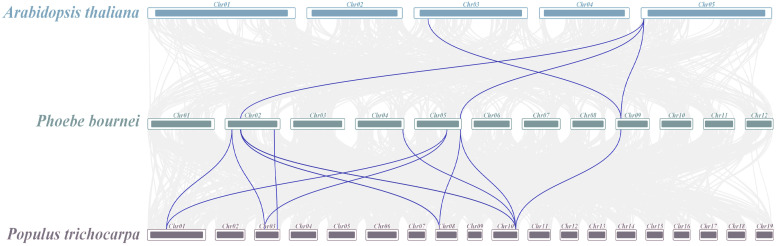
Interspecific covariance among *P. bournei*, *A. thaliana,* and *P. trichocarpa*. Blue lines depict *Hsp70* genes that exhibit covariance across species.

The intraspecific collinearity of the *Hsp70* gene sequence in *P. bournei* was studied, and it was found that there were six pairs of fragments repeat genes in the genome (Figure 5). The collinearity analysis of the *Hsp70* genes from the three plants showed that a total of four *AtHsp70s* were colocalized with *PbHsp70s*, and a total of 11 *PtHsp70s* were colocalized with *PbHsp70s*. The relationship between the *Hsp70* genes and *PtHsp70s* was closer than that between the *Hsp70* genes and *AtHsp70s* in *P. bournei* (Figure 6).

**Figure 7:**
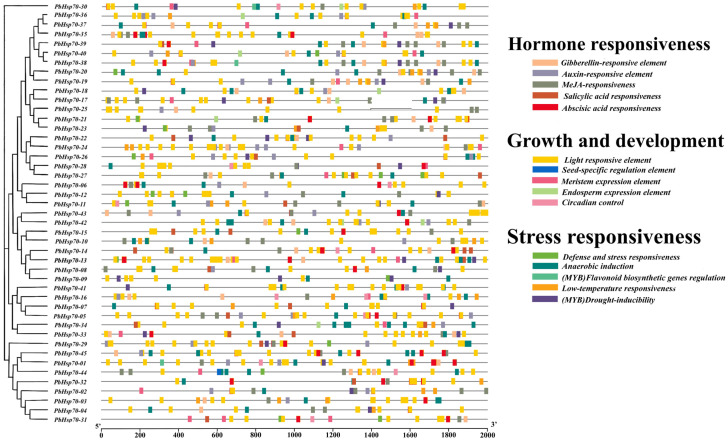
Promoter analysis of the *Hsp70* genes of *P. bournei*.

Two-thousand base-pairs sequences upstream of the coding regions were extracted from the coding regions of all 45 *Hsp70* genes to determine cis-acting regulatory elements (GRES) and to predicate a possible regulatory function of the *Hsp70* genes in *P. bournei* (Figure 7). The *Hsp70* gene family contains multiple types of cis-acting elements that have been implicated in plant hormone responses, growth and development, and abiotic stress responses. Further analysis showed that hormone responsiveness accounted for 34.0%, growth and development accounted for 41.7%, and stress responsiveness accounted for 24.3% of the *PbHsp70* genes. Thus, the cis-responsive elements related to plant growth and development appeared with the highest frequency. This indicates that the *Hsp70* genes play a significant role in plant growth and development under stress conditions. ln addition, the MeJA (methyl jasmonate)-signaling pathway’s responsiveness accounted for 15.0%. MeJA components can have significant effects on plants under heat stress through a variety of pathways [46]. For example, MeJA signaling can activate the expression of genes related to heat-stimulated proteins (Hsps); moreover, by affecting the related protein modification process or interacting with other signaling pathways, MeJA signaling enhances the function of Hsps under heat stress conditions and maintains intracellular protein homeostasis. Thus, it alleviates the cellular damage caused by high temperatures [47]. High-temperature stress leads to the accumulation of reactive oxygen species (ROS) in plants, which can cause oxidative damage to plant cells. The MeJA signaling pathway can induce the expression of various antioxidant enzyme genes, which scavenge excessive ROS in the organism and maintain intracellular redox homeostasis. ABA (Abscisic Acid) is an important adversity hormone that also plays a significant role in the response to heat stress in plants. There is a synergistic effect between the MeJA- and ABA-signaling pathways, which jointly regulate the response to heat stress in plants [48].

### 3.5. Expression Pattern of Hsp70s in P. bournei

**Figure 8:**
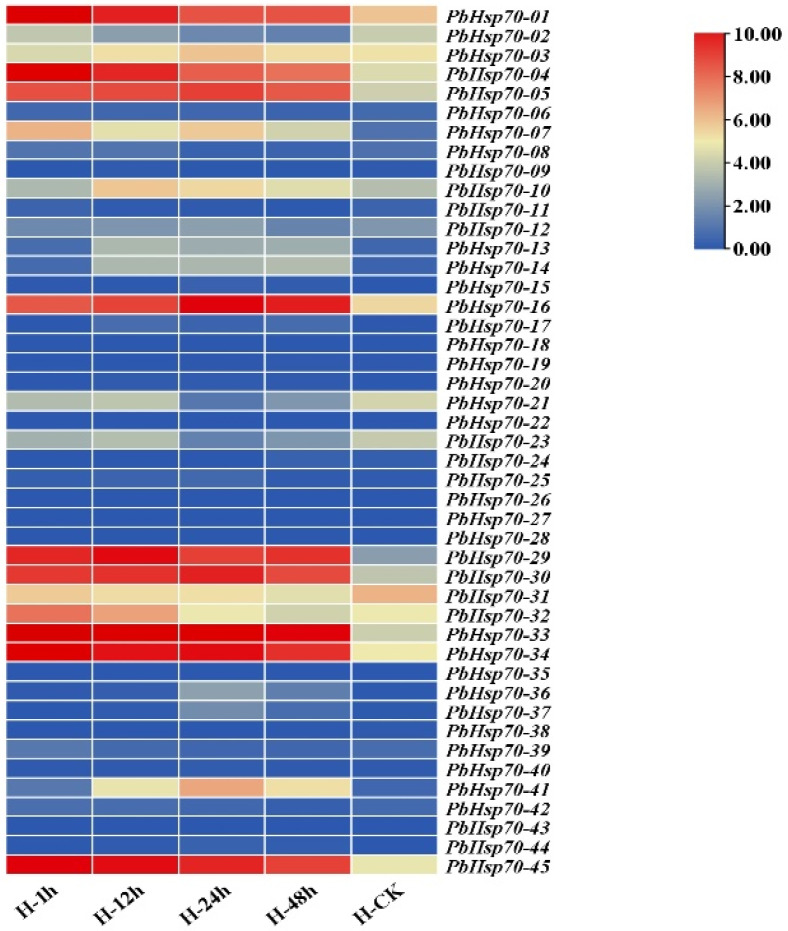
Expression profiles of 45 *PbHsp70* genes. Different colors are used to indicate the expression levels, with the expression values on the right.

A heatmap of the *PbHsp70* family was constructed based on the FPKM values (Figure 8). It showed that the *PbHsp70* family was highly expressed at 12 h. In addition, we found that genes belonging to the same subfamily had resemblance in their expression patterns, with identical expression patterns observed in all three genes of the second subfamily and functionally conserved patterns in the genes of the third subfamily. Over time, the expression of *PbHsp70-45* continued to increase. According to the transcriptome data, genes such as *PbHsp70-33* and *PbHsp70-45* were consistently highly expressed in several samples. Meanwhile, genes such as *PbHsp70-21* and *PbHsp70-23* were highly expressed in the control (H-CK) but significantly downregulated in other samples. These are stress-specific genes, and their expression is precisely regulated by the temperature or other environmental signals. Genes such as *PbHsp70-09* and *PbHsp70-24* were not expressed in most samples. Table S2 shows the FPKM values of *PbHsp70* in the leaves.

### 3.6. RT-qPCR Analysis of Hsp70s in P. bournei

**Figure 9:**
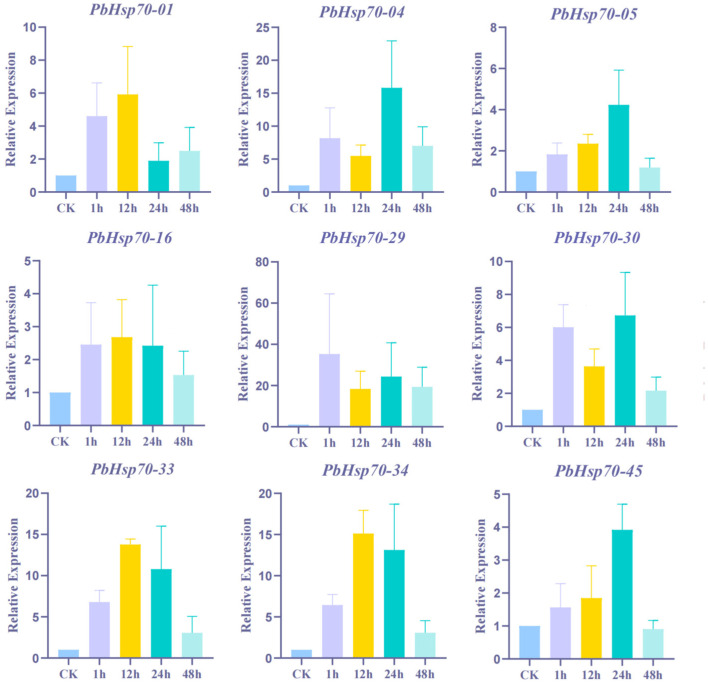
Real-time reverse transcription quantitative PCR (RT-qPCR) validation of nine *Hsp70* genes under high-temperature stress. The y-axis indicates the relative expression value (2^−∆∆CT^) and the x-axis indicates the duration of high-temperature stress. The analysis examined the status of control samples at 0 h before high-temperature stress and then recorded their responses after 1, 12, 24, and 48 h of high-temperature stress. A total of three biological replicates were used in the experiment, each with three technical replicates.

To verify the reliability of the transcriptome data and further investigate the expression patterns of the *Hsp70* genes under heat stress, we selected nine *Hsp70* genes with high expression in *P. bournei* leaves for RT-qPCR analysis. The expression profiles of the nine different genes revealed different degrees of responsiveness to high temperatures (Figure 9). The expression of most genes started to rise after one hour of heat stress, peaked at 24 h and declined again at 48 h, which is consistent with the typical response pattern of heat stress proteins in plants. After undergoing a 24 h period of exposure to elevated temperatures, the samples known as *PbHsp70-04, PbHsp70-05, PbHsp70-30,* and *PbHsp70-45* demonstrated peak levels of expression; then, their levels all decreased to different degrees within 24 h. *PbHsp70-01, PbHsp70-16, PbHsp70-33,* and *PbHsp70-34* were significantly upregulated only in the middle of the pre-stress period, before the initiation of 12 h high-temperature stress. The level of *PbHsp70-29*, which peaked at 24 h, was significantly higher than those of the other genes, and it maintained a high level even at 48 h. However, the expression of *PbHsp70-05* was significantly lower than that of the other members at all time points (Figure 9).

## 4. Discussion

In this study, 45 Hsp70 members were identified in *P. bournei*, which is a greater number than in *A. thaliana* and *P. trichocarpa*. This may be attributed to the fact that *P. bournei* belongs to the family Lauraceae, which has experienced two WGD events and a more complex evolutionary process, and also undergoing a species-specific genome-wide duplication event [49,50]. Gene duplication has significantly contributed to the evolutionary diversity and ecological adaptation of plants, driving phenotypic innovation and functional differentiation [51]. Chromosome segmental duplication and localized tandem duplication constitute two major modes of gene family expansion: the former generates structural variation in chromosomes through non-homologous recombination, and the latter relies on the amplification of neighboring duplication units to form gene clusters [52]. These two replication strategies not only provide plants with the material basis for genetic innovation but also enhance the genome’s capacity to resist stress; this represents an essential force driving the evolution of species [53]. Regarding *P. bournei*, tandem repeat sequence amplification may significantly enhance the plant’s capacity to adapt to environmental fluctuations. It not only provides evolutionary flexibility for gene expression regulation but also serves as an important buffering mechanism to maintain the genetic stability of populations under the pressure of environmental selection [54]. Further studies are necessary to uncover the functions of these duplicated genes and their contributions to phenotypic adaptation in *P. bournei.*

The phylogenetic tree and multiple sequence alignment analysis showed that the *Hsp70* gene family of *P. bournei* could be divided into four subfamilies, I, II, III, and IV, consistent with the classification of the *Hsp70* genes in mosaic bamboo and *Ziziphus jujuba* [55]. The number of genes in subfamily I varies greatly from the number of other subfamilies. It has been shown that the distribution patterns of the motifs among subfamily members correlate with their functional annotation results [56]. An analysis of the conserved motifs present in the *Hsp70* family proteins showed that, despite the significant differences in the conserved motifs between subfamilies, many similar conserved motifs occurred within the same subfamily. This suggests that, despite the variability within this gene family, a certain degree of conservation exists [57]. According to intron-related studies, intron-containing gene architectures exhibit significantly increased sequence lengths and more complex biological function profiles. Such gene architectures may have adaptive advantages during species evolution, especially regarding genome recombination events and the emergence of novel functional genes that show higher plasticity [58,59]. In this study, 77.8% of the genes contained introns, indicating that the *Hsp70* gene family in *P. bournei* retained the typical eukaryotic gene structure during evolution. This intron-rich structure likely represents an evolutionary strategy aimed at balancing functional innovation with genomic stability.

Promoters are the core elements of gene expression regulation; thus, the systematic study of their transcriptional activation properties, epigenetic modification patterns, and environmental response mechanisms is of great theoretical value in revealing cell-specific expression patterns and understanding the mechanisms of gene–environment interactions during the formation of complex traits [60,61]. An array of functional *cis*-elements were identified in the Hsp70 promoter of *P. bournei*, including different categories related to plant growth and development and to light, phytohormones, and stress response factors [62]. Abscisic acid response elements (ABREs) receive abscisic acid molecular signals, thereby regulating the expression of related genes to enhance plants’ resistance to low temperatures, high temperatures, and other types of stress [63,64]. Methyl jasmonate (MeJA) plays an active role in mediating plants’ defense and regulatory responses to stress [65,66]. In this study, for the 45 *Hsp70* genes, we identified several stress-related cis-acting elements in the upstream 2000 bp promoter region. These included abscisic acid response elements, methyl jasmonate response elements, and MYB-binding sites (MYB refers to Myeloblastosis; in plants, MYB transcription factors are proteins that bind to specific DNA sequences and regulate gene expression involved in a wide range of processes), suggesting that the *PbHSP70* family plays an important role in the response to high-temperature stress. It has likely improved the adaptability of *P. bournei* to abiotic stress and significantly contributed to the adaptability and survival of this species in subtropical mountainous climatic zones [67,68].

There is evidence that the *Hsp70* gene family serves critical functions in mediating plants’ thermotolerance mechanisms. For example, in Tausch’s goat grass (*Aegilops tauschii*), under high-temperature stress, members of the *HSP70* gene family enabled enhanced heat tolerance by regulating the antioxidant enzyme system (e.g., Ascorbate Peroxidase (APX) and Superoxide Dismutase (SODase) activity) and chlorophyll synthesis and metabolism, maintaining cell membrane stability and reducing reactive oxygen species accumulation [69]. Meanwhile, in *Beta vulgaris*, the *BvHSP70* gene family was significantly upregulated under high-temperature stress, and its promoter region was enriched with cis-acting elements related to hormone responses, such as abscisic acid and methyl jasmonate response elements, suggesting that this gene family is involved in stress adaptation through hormone-signaling pathways [70]. In this study, we observed the differential response characteristics of the *Hsp70* gene family in *P. bournei* under heat stress. The expression levels of most of the *PbHsp70* family were significantly increased, suggesting that the *PbHsp70* genes are responsive to high-temperature stress, and that high temperatures induced the expression of some of them, while repressing their expression. According to the expression profile of the *PbHsp70* genes, genes with high expression may be responsible for basal stress tolerance and serve as constitutive stress tolerance genes. Some genes were not expressed in all samples; these may be pseudogenes or redundant backup genes that were retained in evolution but are activated only under extreme conditions [71]. According to the transcriptome data, *PbHsp70-29* may be a key gene in the response to high-temperature stress in *P. bournei*. This gene is responsible for coordinating the co-expression of other stress-resistant genes. Its high expression may be related to the substrate-binding ability or stability [72]. Meanwhile, the expression level of *PbHsp70-05* was significantly lower than that of other members at all time points. This may be a redundant gene or a functional differentiation gene that plays a role in specific stress types but is not activated under high-temperature stress. Further analysis revealed that most of the genes that were upregulated under high-temperature stress belonged to subfamily II rather than subfamily III. The members of this subfamily maintained high expression after sustained high-temperature exposure, suggesting that they may be involved in the regulation of cellular thermal homeostasis through the mechanisms of conformational stability and the maintenance of ATPase activity. This indicates that these two families have strong potential for heat tolerance [73]. In conclusion, this study highlights the differential expression and potential roles of the *PbHsp70* gene family in response to high-temperature stress in *P. bournei*. The findings provide valuable insights into this family’s regulatory mechanisms and several key genes involved in heat tolerance. In future studies, we will clarify whether *Hsp70* is a hub gene by studying the synergistic response pattern of the *Hsp70* family under combined stress, which may contribute to breeding programs aimed at enhancing plants’ heat stress resistance.

## 5. Conclusions

In this research, the *Hsp70* gene family of *P. bournei* was characterized. For the first time, a total of 45 *PbHsp70* genes were identified and analyzed with respect to their physicochemical properties, conserved motifs, and exon–intron structures. These *Hsp70* genes were found to be highly conserved. We also analyzed the chromosomal localization of *P. bournei* and performed an interspecific covariance analysis of its *Hsp70* genes and those of *A. thaliana* and *P. tremula*, providing valuable biological information about the evolutionary relationships of *P. bournei*. The process of identifying *cis-*acting elements within the promoter region of the *Hsp70* genes contributes to revealing the pathways involved in *P. bournei*’s response to abiotic stress. The expression of the *Hsp70* genes was investigated at five high-temperature stages, and the expression patterns in the leaves were verified. It was found that eight *Hsp70* genes (*PbHsp70-01, PbHsp70-04, PbHsp70-05, PbHsp70-29, PbHsp70-30, PbHsp70-33, PbHsp70-34,* and *PbHsp70-45*) have potential roles in the heat stress response. These observations not only deepen our knowledge of the functional roles of this gene family but also provide a theoretical basis for the study of stress tolerance in this plant species.

## Figures and Tables

**Table 1:** Physicochemical properties of Hsp70 proteins from *P. bournei*.

Member	Amino Acid (aa)	Grand Average of Hydrophobicity	Theoretical pI	Molecular Weight	Aliphatic Index	Instability Index	Subcellular Localization
PbHsp70-01	663	−0.456	5.12	73,265.1	87.95	27.01	E.R.
PbHsp70-02	803	−0.428	5.6	89,802.35	79.44	51.6	nucl
PbHsp70-03	835	−0.441	5.24	92,764.05	78.12	49.06	cyto
PbHsp70-04	859	−0.454	5.33	95,252.16	77.53	44.57	chlo
PbHsp70-05	585	−0.416	5.11	64,506.31	84.72	35.09	cyto
PbHsp70-06	555	−0.326	5.67	62,187.26	93.64	36.96	cyto
PbHsp70-07	232	−0.782	4.74	25,677.78	74.48	32.83	mito
PbHsp70-08	155	−0.794	5.29	17,910.22	80.58	44.47	nucl
PbHsp70-09	678	−0.592	8.97	75,778.14	80.74	53.8	nucl
PbHsp70-10	117	−1.119	6.19	13,829.69	73.5	49.48	cyto
PbHsp70-11	201	−0.791	6.86	22,829.8	76.27	56.09	cyto
PbHsp70-12	304	−0.436	5.96	34,209.25	90.76	30.39	cyto
PbHsp70-13	407	−0.379	8.73	46,087.08	83.69	34.91	chlo
PbHsp70-14	226	−0.648	5.08	25,399.72	82.92	31.63	cysk
PbHsp70-15	215	−0.396	8.56	24,200.87	83.49	32.97	chlo
PbHsp70-16	651	−0.414	5.05	71,339.71	83.01	32.89	cyto
PbHsp70-17	292	−0.446	6.98	32,504.12	90.86	35.27	cyto
PbHsp70-18	166	−0.743	9.06	18,816.27	78.8	36.09	nucl
PbHsp70-19	156	−0.401	6.16	18,000.74	103.21	30.24	cyto/nucl
PbHsp70-20	135	−0.457	5.87	15,406.6	90.96	43.72	cyto/nucl
PbHsp70-21	119	−0.306	9.25	12,926.8	87.56	29.35	cyto
PbHsp70-22	165	−0.520	9.49	18,538.57	88.79	40.07	mito
PbHsp70-23	327	−0.385	7.69	36,460.54	87.68	30.26	chlo
PbHsp70-24	114	−1.011	9.71	13,319.34	67.72	46.18	nucl
PbHsp70-25	580	−0.481	5.39	65,468.48	88.93	27.88	golg
PbHsp70-26	146	−0.546	6.18	16,641.12	91.58	42.02	cyto
PbHsp70-27	319	−0.609	9.56	35,633.63	75.86	35.61	chlo
PbHsp70-28	750	−0.308	9.18	85,255.63	89.36	42	vacu
PbHsp70-29	366	−0.613	4.95	39,992.95	75.96	32.29	cyto
PbHsp70-30	653	−0.397	5.13	71,554.09	83.52	32.98	cyto
PbHsp70-31	858	−0.414	5.23	94,470.99	79.69	42.68	chlo
PbHsp70-32	889	−0.417	5.48	98,969.28	87.53	38.38	E.R.
PbHsp70-33	620	−0.384	4.9	67,770.73	84.18	33.81	cyto
PbHsp70-34	554	−0.392	8.78	61,513.15	83.59	39.59	cyto
PbHsp70-35	257	−0.526	6.36	28,915.68	83.19	23.18	cyto
PbHsp70-36	676	−0.365	9.25	76,069.63	92.72	32.83	cyto
PbHsp70-37	291	−0.477	9.75	33,607.19	99.14	27.06	cyto
PbHsp70-38	182	−0.417	9.3	21,032.39	101.87	25.20	cyto
PbHsp70-39	183	−0.455	9.19	21,087.38	100.77	23.37	cyto
PbHsp70-40	660	−0.321	7.18	73,827.46	93.95	32.84	cyto
PbHsp70-41	649	−0.406	5.14	71,262.93	82.97	33.84	cyto
PbHsp70-42	1358	−0.308	7.94	150,806.92	86.48	38.61	chlo
PbHsp70-43	515	−0.752	4.65	57,613.73	74.08	42.83	cyto
PbHsp70-44	425	−0.770	4.81	47,137.06	76.66	41.63	cyto
PbHsp70-45	652	−0.494	5.26	71,897.49	86.01	27.7	golg

Note: E.R: Endoplasmic reticulum; nucl: Nucleus; cyto: Cytoplasmic; chlo: Chloroplast; mito: Mitochondrial; cysk: cytoskeleton; golg: Golgi body; vacu: Vacuole.

## Data Availability

The original data presented in the study are openly available in the. National Center for Biotechnology Information (NCBI, https://www.ncbi.nlm.nih.gov/, accessed on 18 January 2025) genome database.

## Supplementary Materials

The following supporting information can be downloaded at: https://www.mdpi.com/article/10.3390/biology14060602/s1, Table S1. qRT-PCR primers. Table S2. The FPKM values of *PbHsp70* in the leaves.
