# Supplementary material for: Genome-Wide Identification and Heat Stress-Induced Expression Profiling of the Hsp70 Gene Family in Phoebe bournei
Source: Biology (Basel). 2025 May 25;14(6):602. doi: 10.3390/biology14060602 (PMC12189233; doi:10.3390/biology14060602)
Supplement: Supplementary file 1 [file biology-14-00602-s001.zip › Table S1 qRT-PCR primers.pdf]

Table S1 qRT-PCR primers

Gene name	Gene ID		Primers(5' to 3')
PbHsp70-01	OF26547	F	CTTGGTATTGAGACTGTTGGTGGAG
		R	ACGGTCTGCTGATCCTGGTAAG
PbHsp70-04	OF04365	F	AAGAGGAAGGCTGAGACACTGG
		R	TCGGTGGCTTGCTGTGGAG
PbHsp70-05	OF19069	F	AGAGGCTTATCTTGGCTCCACTG
		R	AACCAGCAATAACACCAGCATCC
PbHsp70-16	OF19044	F	GGTTGGAGACTGCTGGAGGTG
		R	GGCTGGTTGTCCGAGTAGGTAG
PbHsp70-29	OF07344	F	GAGGAATACTGTGAAGGACGAGAAG
		R	CCTATCCAACCACTCAATCACTTCC
PbHsp70-30	OF01959	F	AAGATTACCATCACCAACGACAAGG
		R	TTCTTCCTGTGCTCCTCATCCTC
PbHsp70-33	OF05254	F	AAGAATGCTGTGGTCACTGTTCC
		R	GGCTGTAGGCTCGTTGATGATAC
PbHsp70-34	OF05259	F	TTAAGAATGCCGTGGTCACTGTTC
		R	GGCTGTAGGCTCGTTGATGATAC
PbHsp70-45	OF00056	F	GAAGCCGAGCGTGCCAAGAG
		R	GCAGCCCAGCATCGTCCATTG
Internal reference		F	CATTCAAGTATGCGTGGGT
		R	ACGGTGACCAGGAGCA
