# Supplementary material for: Genome-Wide Identification and Heat Stress-Induced Expression Profiling of the Hsp70 Gene Family in Phoebe bournei
Source: Biology (Basel). 2025 May 25;14(6):602. doi: 10.3390/biology14060602 (PMC12189233; doi:10.3390/biology14060602)
Supplement: Supplementary file 1 [file biology-14-00602-s001.zip › Table S2 The FPKM values of PbHsp70 in the leaves.pdf]

ID	Rename	H_1h	H_12h	H_24h	H_48h	CK
0F26547	*PbHsp70-01*	3128.06253	1054.046467	422.501767	477.2095	59.6992667
0F19734	*PbHsp70-02*	13.0818	4.512033333	2.16836667	1.74206667	14.7783667
0F07161	*PbHsp70-03*	5.1977	7.122533333	5.29516667	4.00383333	4.41263333
0F04365	*PbHsp70-04*	1991.3788	994.7799333	331.851667	262.9555	21.4122333
0F19069	*PbHsp70-05*	423.314833	461.4053333	548.798033	366.055533	16.2817
0F19066	*PbHsp70-06*	0.54226667	0.4636	0.41483333	0.32926667	0.53676667
0F19061	*PbHsp70-07*	80.9746667	42.5641	65.7351	25.4621333	0.91096667
0F19058	*PbHsp70-08*	1.1911	0.968633333	0.30013333	0.3154	0.83576667
0F19057	*PbHsp70-09*	0.0108	0.0873	0.01753333	0.0501	0.05873333
0F19056	*PbHsp70-10*	10.2970333	69.3866	44.2727333	53.3222667	10.3319667
0F19055	*PbHsp70-11*	0.3924	0.094833333	0.05876667	0.02613333	0.34866667
0F19054	*PbHsp70-12*	2.32436667	3.405566667	4.5995	1.92883333	3.36643333
0F19053	*PbHsp70-13*	0.66323333	11.71663333	7.10733333	12.1526	0.43873333
0F19052	*PbHsp70-14*	0.60686667	10.76463333	9.24776667	12.7166333	0.367
0F19051	*PbHsp70-15*	0.04013333	0.033333333	0.29406667	0.1318	0
0F19044	*PbHsp70-16*	399.485133	525.9152667	1556.57073	993.7983	43.3759
0F19043	*PbHsp70-17*	0	0.779066667	0.37453333	0.70913333	0.02413333
0F19042	*PbHsp70-18*	0	0	0	0	0
0F19041	*PbHsp70-19*	0	0	0.02303333	0.05983333	0.0401
0F19040	*PbHsp70-20*	0	0.112533333	0.08756667	0.07793333	0
0F19039	*PbHsp70-21*	9.67923333	16.68316667	1.89666667	3.42776667	18.3568
0F19038	*PbHsp70-22*	0	0	0	0	0
0F19035	*PbHsp70-23*	7.24046667	12.40483333	2.13853333	3.52896667	14.2624667
0F19034	*PbHsp70-24*	0	0	0	0.26606667	0.17233333
0F19031	*PbHsp70-25*	0.1722	0.3232	0.6424	0.0794	0.08766667
0F19028	*PbHsp70-26*	0	0	0	0	0
0F19027	*PbHsp70-27*	0	0	0	0	0
0F19026	*PbHsp70-28*	0	0.010166667	0.01583333	0.0141	0
0F07344	*PbHsp70-29*	833.071567	2396.486367	553.432967	866.760567	4.24356667
0F01959	*PbHsp70-30*	670.489767	713.4324667	945.025733	512.1646	12.2418667
0F11103	*PbHsp70-31*	55.0179	39.7213	37.4705333	24.1358667	78.4085333
0F05133	*PbHsp70-32*	270.8928	108.1350667	30.4207333	17.7460667	29.9009667
0F05254	*PbHsp70-33*	7895.533	4918.607333	3091.21293	1782.8613	16.3132333
0F05259	*PbHsp70-34*	3391.1263	1428.0467	1346.31447	860.740333	31.5177667
0F18261	*PbHsp70-35*	0	0	0	0	0
0F18236	*PbHsp70-36*	0.0654	0.1764	5.57836667	1.7799	0.03553333
0F17949	*PbHsp70-37*	0	0.104433333	3.146	0.9491	0.0546
0F17944	*PbHsp70-38*	0	0	0	0.02886667	0.06043333
0F17942	*PbHsp70-39*	1.25683333	0.602366667	0.46086667	0.4986	0.66666667
0F17933	*PbHsp70-40*	0.07383333	0.0673	0.12036667	0.0588	0.05656667
0F02766	*PbHsp70-41*	1.64223333	27.7637	98.7605667	42.8231667	0.41643333
0F02769	*PbHsp70-42*	0.71733333	0.667366667	0.46076667	0.1944	0.5105
0F02770	*PbHsp70-43*	0	0	0	0	0
0F06376	*PbHsp70-44*	0.02756667	0.101066667	0.2495	0.17216667	0
0F00056	*PbHsp70-45*	1739.1496	1330.936233	984.3512	547.799267	27.5765667
